# Introduction of an isoxazoline unit to the β-position of porphyrin via regioselective 1,3-dipolar cycloaddition reaction

**DOI:** 10.3762/bjoc.15.143

**Published:** 2019-06-28

**Authors:** Xiujun Liu, Xiang Ma, Yaqing Feng

**Affiliations:** 1 Research Center of Analysis and Test, School of Chemistry and Molecular Engineering, East China University of Science & Technology, Meilong Road 130, Shanghai 200237, China; 2 School of Chemical Engineering and Technology, Tianjin University, Tianjin 300072, China; 3 Collaborative Innovation Center of Chemical Science and Engineering, Tianjin 300072, China

**Keywords:** dipolar cycloaddition, isoxazoline, macrocycles, nitrile oxide, porphyrin

## Abstract

Isoxazoline-linked porphyrins have been synthesized by a regioselective 1,3-dipolar cycloaddition reaction between vinylporphyrin **2** and nitrile oxides. The steric interaction directed the reaction trajectory, in which only the product with a link between the 5-position of the isoxazoline and the β-position of porphyrin was observed. The isoxazoline-porphyrins **3a**,**b** have been characterized by absorption, emission, ^1^H NMR and mass spectra. Later, the crystal structure of **3a** was obtained and confirmed the basic features of the NMR-derived structure. Furthermore, a pair of enantiomers of **3a** presented in the crystal, which formed a dimeric complex through intermolecular coordination between the Zn^2+^ center and the carbonyl group of the second molecule.

## Introduction

In nature, porphyrin-type compounds play a prominent role in life [1]. It is well known that certain vital functions, like O_2_ transport, photosynthesis etc. depend on the action of porphyrin–metal complexes [2–5]. Inspired by the natural porphyrinoids, some man-made porphyrinoids have been designed to mimic the characteristic functions with purposes of utilization in various fields (please see recent reviews [6–14]). In order to achieve application-oriented molecules, lots of modification approaches have been developed [15–24]. Among them, the 1,3-dipolar cycloaddition reaction [25] is an efficient method to fuse five-membered rings on the periphery of the porphyrin framework. Because the periphery double bonds of the porphyrin macrocycle are nice dipolarophiles, and can trap 1,3-dipoles to furnish the chlorin or bacteriochlorin analogues [26–37]. On the other hand, the formed heterocycles are also very important. For example, isoxazoline derivatives are not only significant intermediates in organic synthesis, e.g., masked aldols [38–39], but also have a broad spectrum of interesting bioactivities, e.g., anti-inflammatory [40–41]. In addition, a few porphyrin-based dipoles have been also reported [42]. Recently, a 1,3-dipolar cycloaddition reaction was performed between the vinyl group at methyl pheophorbide A and the in-situ-generated nitrile oxide, which showed inferior selectivity and afforded regio-/stereoisomers [43]. NMR analysis assisted the identification of various isomeric isoxazoline-linked chlorin products. Here, we would like to report an artificial vinylporphyrin **2**, which has been designed to control the regioselectivity and reaction trajectory of the 1,3-dipolar cycloaddition reaction by steric hindrance. The obtained novel isoxazoline-substituted porphyrin derivatives **3a**,**b** have been characterized by absorption, emission, NMR and mass spectrometry. In addition, the crystal structure of **3a** is discussed.

## Results and Discussion

**Scheme 1:**
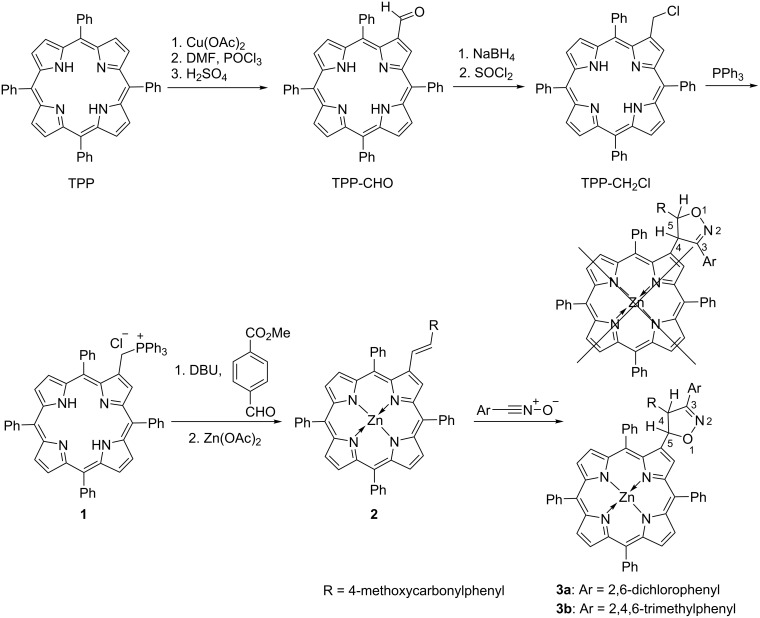
Synthetic route of β-isoxazoline linked porphyrin **3**.

The synthetic route to the β-isoxazoline-substituted porphyrin is depicted in Scheme 1, in which the double bond was furnished by Wittig reaction, followed by 1,3-dipolar cycloaddition reaction with stable dipoles. In detail, tetraphenylporphyrin (TPP) was used as starting material. A Vilsmeier reaction was carried out after insertion of Cu^2+^ into the cavity of TPP. In the presence of concentrated H_2_SO_4_ the Cu^2+^ was removed to give the 2-formyl derivative TPP-CHO. Subsequently, the formyl group was reduced by NaBH_4_, accompanied with chlorination by SOCl_2_, to afford the chloromethyl derivative TPP-CH_2_Cl. Notably, this intermediate was labile on silica-gel column to give the precursor hydroxymethylporphyrin. Hence recrystallization was employed to purify it. Later, refluxing of the solution of TPP-CH_2_Cl and PPh_3_ in toluene gave the phosphonium salt **1** [44]. To introduce the vinyl group at the β-position, a Wittig reaction was performed, in which the porphyrin phosphonium salt **1** reacted with 4-MeCO_2_-benzaldehyde in the presence of DBU to furnish the double bond, followed by insertion of Zn ions into the porphyrin cavity to give compound **2**. Nitrile oxides [45] as one of the most reactive dipoles not only react with various dipolarophiles but also undergo spontaneous dimerization to form furoxans. To avoid or minimize this drawback, the substitutents (e.g., Cl or Me) around the CNO group were introduced to stabilize the nitrile oxides even at room temperature [46–47]. Here, the 2,6-dichlorophenyl- or 2,4,6-trimethylphenyl nitrile oxides were used to react with **2** at 110 °C for 12 hours in toluene. Generally, two products with different positions (C4 or C5) of isoxazoline connected to the β-position of porphyrin should be obtained if the stereochemistry was not considered (Scheme 1) [43]. However, only one fraction was isolated in this reaction besides the unconsumed starting material **2**.

**Figure 1:**
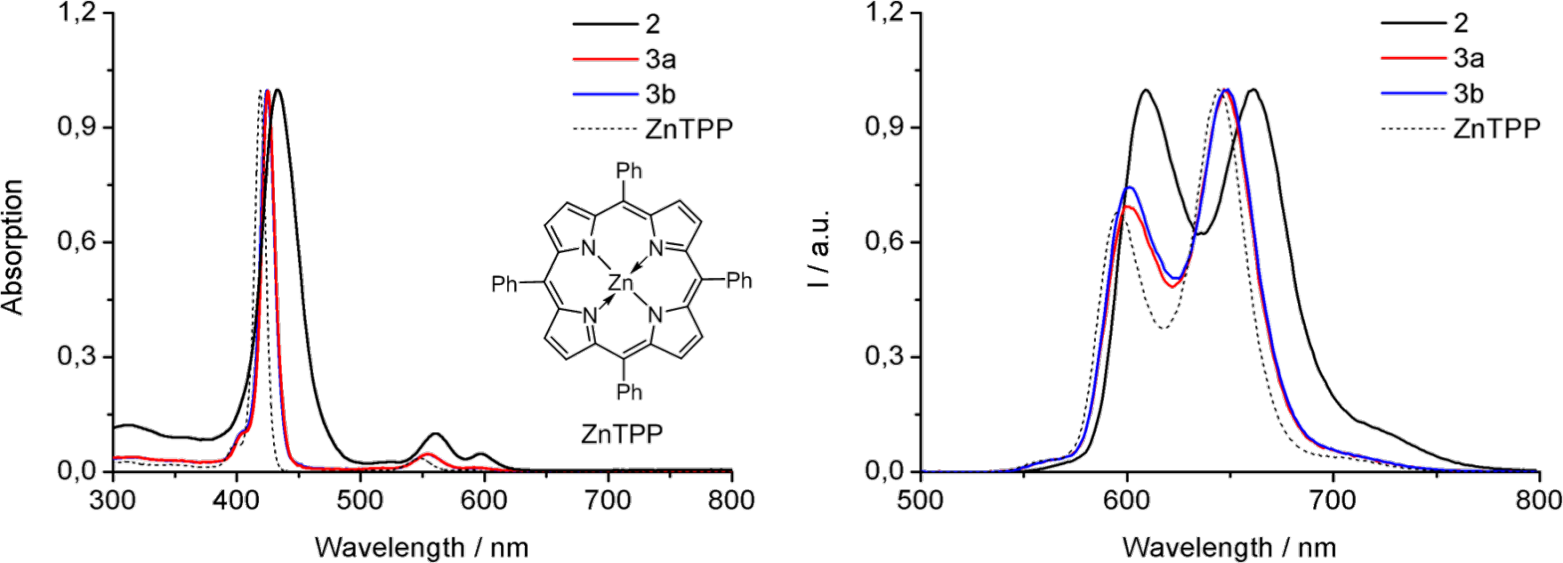
Normalized UV–vis and emission spectra of β-isoxazoline porphyrins **3a**,**b**, **2** and ZnTPP in CH_2_Cl_2_.

The structure of 1,3-dipole cycloaddition products **3a**,**b** were firstly characterized by absorption spectroscopy (Figure 1). The two compounds **3a**,**b** showed approximately overlapped traces with typical intense Soret band and two weak Q bands. When compared to the spectrum of the precursor **2**, a blue shift of ≈10 nm was observed in those of **3a** or **3b**, which may result from the loss of partial conjugation after the cycloaddition reaction between the nitrile oxides and C=C double bond. However, the substitution at the β-position in **3a** or **3b** led to a ≈5 nm red shift when compared with the absorption spectrum of ZnTPP [48]. In addition, the fluorescence spectra of **3a** and **3b** were comparable with that of ZnTPP, and again shifted to short wavelength (≈14 nm) with respect to that of **2**. Later the ESIMS spectrum confirmed the constitution of two compounds, respectively. The pseudo molecular ions were observed at *m*/*z* = 1024.1765, demonstrating the molecular formula of C_61_H_39_Cl_2_N_5_O_3_Zn for **3a**, and *m*/*z* = 998.3011, indicating the molecular formula of C_64_H_47_N_5_O_3_Zn for **3b**. Thus, the formal formulas of **3a**,**b** are the sum of porphyrin **2** and the corresponding nitrile oxides.

**Figure 2:**
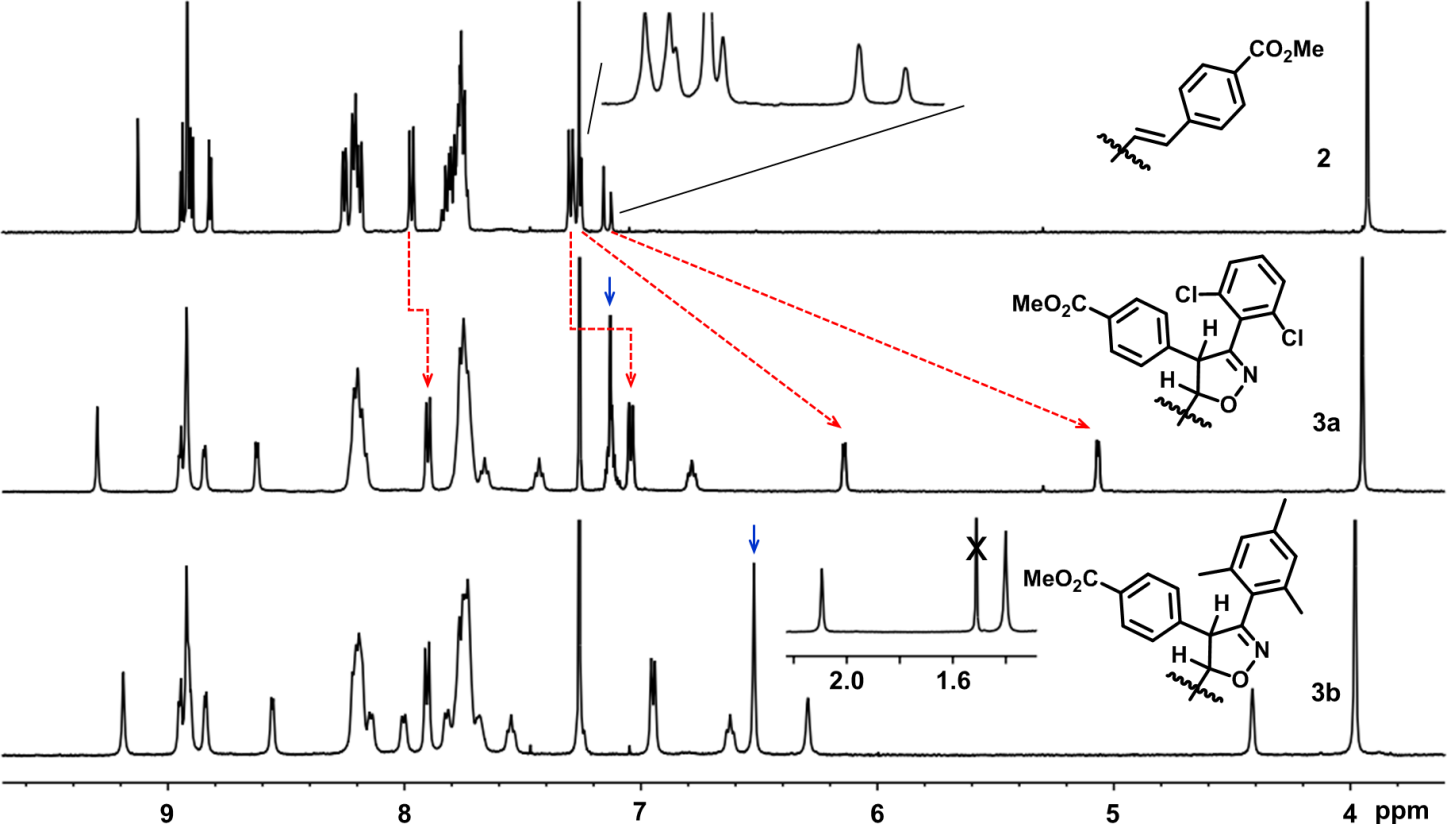
The ^1^H NMR spectra of **2** (top, partially zoomed around 7.26 ppm), **3a** (middle, red arrows indicate the high field shift of the protons after 1,3-diploar cycloaddition, while the blue one is the signal of aromatic protons of the dichlorophenyl group) and **3b** (bottom, inset is the signal of CH_3_ around 2 ppm, x marks the signal of H_2_O, the blue one is the signal of aromatic protons on trimethylphenyl group) in CDCl_3_ (500 MHz, 25 °C).

Subsequently, the analysis of the ^1^H NMR spectra helped to establish the structure of compound **3a**,**b** (Figure 2). In the ^1^H NMR spectrum of **2**, the signals of the vinyl group were observed at 7.15 ppm (*J* = 16.0 Hz) and 7.28 ppm (*J* = 16.0 Hz, Figure 2, top), indicating a *trans*-configuration of the double bond. The signal of the CO_2_Me group occurred at 3.93 ppm, while those of the β-pyrrolic protons were in the range of 8.82 ppm to 9.13 ppm.

As consequence of the cycloaddition reaction, the signals of the vinyl group vanished in the spectra of **3a** or **3b**. Taking the analysis of **3a** as example. Two doublets at 5.07 ppm (*J* = 4.0 Hz) and 6.15 ppm (*J* = 4.0 Hz) aroused, which should be assigned to the two saturated CHs after dipolar cycloaddition reaction between the vinyl group and dichlorophenyl nitrile oxides. A multiplet was observed around 7.13 ppm and assigned to the aromatic protons of the 2,6-dichlorophenyl group. Meanwhile the aromatic signals of C_6_H_4_CO_2_Me shifted to the higher field when compared to that of **2**, owing to the disturbing of the de-shielding effect from the double bond and porphyrin framework. Similar variation was also realized in the spectrum of **3b**. Based on these, the cycloaddition reaction should take place on the β-vinyl group but not the double bond on the porphyrin framework, indicating the high activity of the β-vinyl group over the double bond on the macrocycle. However, at which position of the newly formed isoxazoline is linked to the porphyrin is still elusive.

**Figure 3:**
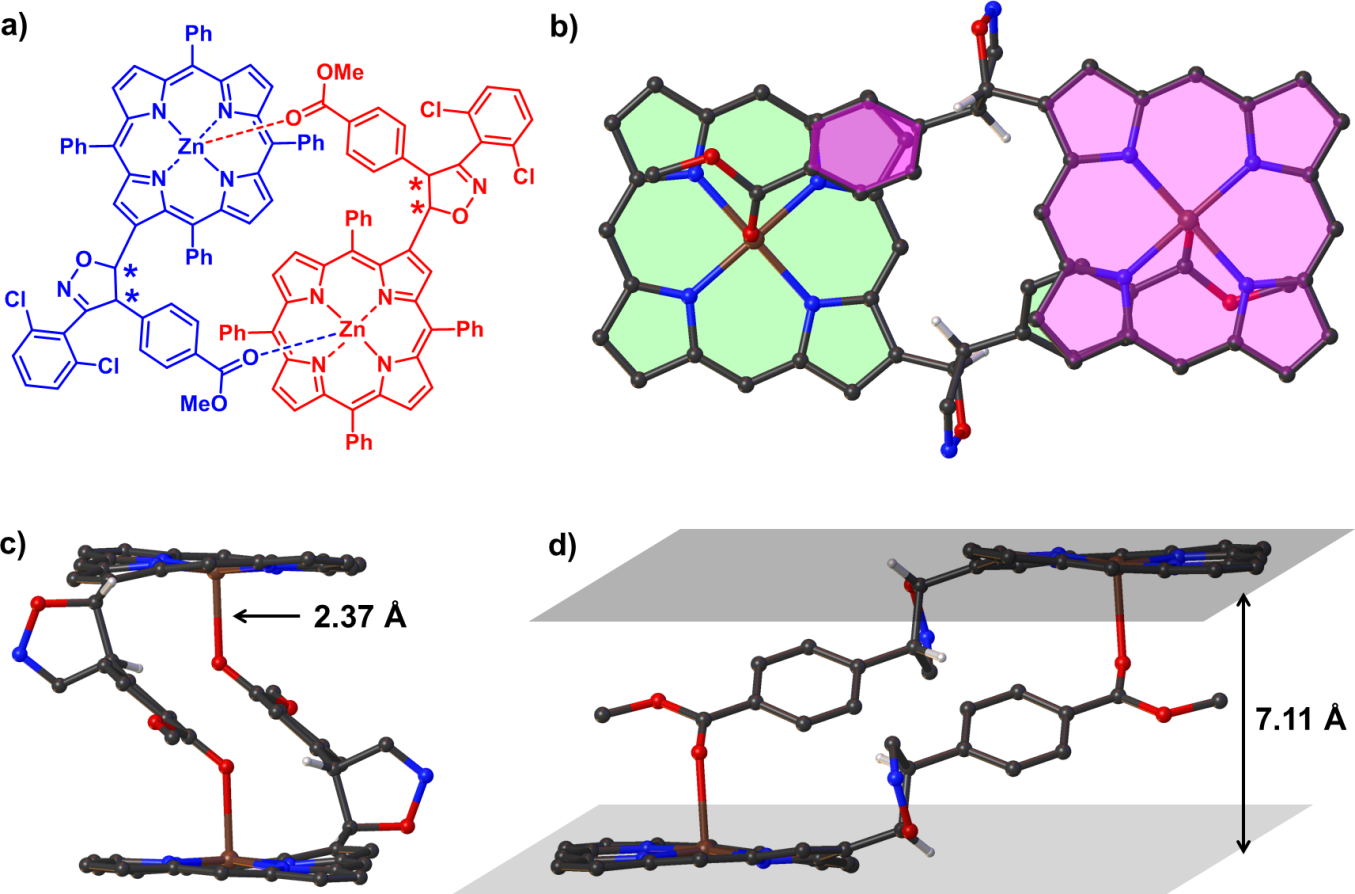
The representative dimeric structure of **3a** according to the crystal structure (a) and enantiomeric dimer structure in crystal (b–d, the phenyl group at *meso*-positions and 2,6-dichlorophenyl group were omitted for the sake of clarity). The distances of Zn–O (c) and distance between the two mean planes of porphyrins (d) has been labeled.

Later, a single crystal of **3a** was obtained by diffusion of *n*-hexane to the solution of **3a** in CHCl_3_ at 23 °C. The structure of **3a** was unambiguously established by single-crystal X-ray diffraction analysis. Compound **3a** crystallized in the monoclinic with space group *C*2/*c*. Figure 3 shows the dimeric structure of **3a** (front view and side view). Notably, only the 5-position of isoxazoline linking to porphyrin was observed. As expected, the steric clash of the substituent on nitrile oxide and porphyrin framework seems to confine the reaction trajectory and direct nitrile oxide to approach the double bond from outside the macrocycle. In addition, a pair of enantiomers is revealed which formed a dimeric fashion with the help of the fifth coordination to the Zn^2+^ by the carbonyl group from the other molecule. The distance of Zn–O is found to be 2.37 Å, which is longer than the distance when MeOH coordinated to the Zn^2+^ ions [49]. This may attribute to the larger volume of the C_6_H_4_CO_2_Me group than MeOH when closing to the Zn^2+^ center. In the dimer structure, two planes defined by porphyrin macrocycles are parallel with a distance of 7.11 Å. The bulky substituent group around the β-position, in conjunction with the presence of four benzene rings attached to the central porphyrin core, twist the overall geometry of the molecule. For instance, the porphyrin core is slightly distorted with deviation of 0.14 Å. The dihedral angles ranges from 2.6° to 8.7° for the four pyrrole rings with respect to the porphyrin mean plane, which are more twisted than that in normal Zn-porphyrin [49]. The lengths of Zn–N bonds range from 2.041 Å to 2.073 Å. The new formed five-membered isoxazoline ring is almost vertical (with a dihedral angle of 82.7°) to the mean plane of the porphyrin core. While the dihedral angle between the plane defined by macrocycle and the methoxycarbonylbenzene ring was found to be 40.5°.

## Conclusion

In summary, two novel isoxazoline-substituted porphyrin derivatives **3a**,**b** have been synthesized via the regiospecific and steric-oriented 1,3-dipolar cycloaddition reaction of vinylporphyrin **2** and two nitrile oxides, respectively. Only the product with a link between the 5-position of the isoxazoline and the β-position of porphyrin was obtained, directed by steric hindrance when the two components are approaching to each other. Owing to the interruption of the double bond after the cycloaddition reaction, the absorption spectrum shifted to shorter wavelengths. The isoxazoline-modified porphyrins have been identified by absorption, emission, ^1^H NMR and mass spectrometry, and **3a** was further characterized by X-ray analysis. In the crystal, a pair of enantiomers of **3a** is present, which assembled to a dimeric structure with the fifth chelation of a Zn^2+^ ion by the carbonyl group of the other molecule. The self-dimerization property of **3a** may be utilized in supramolecular chemistry [50–53].

## Experimental

**1**: Porphyrin phosphonium salt **1** was prepared according to the literature [44], the yield was 51%. ^1^H NMR (500 MHz, CDCl_3_) δ –2.77 (s, 2H, inner NH), 5.19–5.22 (d, *J* = 15.0 Hz, 2H, CH_2_P), 7.10–7.14 (m, 6H), 7.27–7.30 (m, 5H), 7.39 (d, *J* = 10.0 Hz, 2H), 7.40–7.89 (m, 18H), 8.17–8.19 (m, 4H), 8.31 (d, *J* = 4.0 Hz, 1H, β-pyrrolic H), 8.46 (d, *J* = 4.0 Hz, 1H, β-pyrrolic H), 8.76–8.86 (m, 5H, β-pyrrolic H).

**2**: To the solution of compound **1** (0.10 g, 0.11 mmol) and 4-methoxycarbonylbenzaldehyde (18 mg, 0.11 mmol) in CH_2_Cl_2_ (20 mL) was added DBU (83 mg, 0.55 mmol, 5.0 equiv vs **1**). The reaction mixture was stirred for 8 hours. H_2_O (15 mL) was added to quench the reaction. The organic phase was separated and dried with MgSO_4_, followed by filtration and concentration to obtain a dark red residue. The product was isolated as first fraction by silica gel column chromatography with an eluent consisting of CH_2_Cl_2_/petrol ether 1:1 (v:v). Finally, the free-base porphyrin **2-H_2_** was obtained in a yield of 68 mg (82%). The obtained free-base porphyrin **2-H_2_** (50 mg, 0.065 mmol) was dissolved in CHCl_3_ (20 mL). Then the solution of Zn(OAc)_2_·2H_2_O (28.5 mg, 0.13 mmol, 2 equiv) in MeOH (6 mL). After 2 hours, TLC analysis showed the completion of the reaction. H_2_O was added to quench the reaction and the organic phase was collected. Then the organic phase was dried by MgSO_4_, followed by filtration and concentration under reduced pressure. The raw product was purified by silica gel column chromatography with eluent of CH_2_Cl_2_/petrol ether 3:1 (v:v). Finally, compound **2** was obtained in a yield of 92% (50.2 mg). ^1^H NMR (500 MHz, CDCl_3_) δ 3.93 (s, 3H, CO_2_CH_3_), 7.15 (d, *J* = 16.0 Hz, 1H, CH=CH), 7.28 (d, *J* = 16.0 Hz, 1H, CH=CH), 7.30 (d, *J* = 8.0 Hz, 2H), 7.75–7.85 (m, 12H), 7.97 (d, *J* = 8.0 Hz, 2H), 8.18–8.22 (m, 6H), 8.24–8.26 (m, 2H), 8.83 (d, *J* = 4.0 Hz, 1H, β-pyrrolic H), 8.90 (d, *J* = 4.0 Hz, 1H, β-pyrrolic H), 8.91–8.92 (m, 3H, β-pyrrolic H), 8.94 (d, *J* = 4.0 Hz, 1H, β-pyrrolic H), 9.13 (s, 1H, β-pyrrolic H); ESIMS *m*/*z*: [M + H]^+^ calcd for C_54_H_39_N_4_O_2_ZnF, 837.2; found, 837.1; UV–vis (CH_2_Cl_2_) λ_max_: 432, 561, 597 nm.

**3a**: A solution of **2** (100 mg, 0.12 mmol) and nitrile oxide (0.6 mmol, 5 equiv) in dry toluene (20 mL) was heated to reflux for 6 hours under N_2_. Subsequently, addition of two portions of 2,6-dichlorophenyl nitrile oxide [45] (0.3 mmol) were added to the reaction mixture every 3 hours. The reaction mixture was concentrated to dryness under reduced pressure and the product was isolated by silica gel column chromatography with eluent of DCM/ethyl acetate 50:1 (v/v). The second purple fraction was collected. Finally, **3a** was obtained in a yield of 48% (59 mg) as purple powder. ^1^H NMR (500 MHz, CDCl_3_) δ 3.95 (s, 3H, CO_2_CH_3_), 5.07 (d, *J* = 4.0 Hz, 1H, CH), 6.15 (d, *J* = 4.0 Hz, 1H, CH), 6.79 (t, *J* = 6.0 Hz, 1H), 7.05 (d, *J* = 8.0 Hz, 2H), 7.12–7.15 (m, 3H), 7.44 (t, *J* = 6.0 Hz, 1H), 7.67 (t, *J* = 6.0 Hz, 1H), 7.76–7.77 (m, 10H), 7.91 (d, *J* = 8.0 Hz, 2H), 8.17–8.22 (m, 7H), 8.63 (d, *J* = 4.0 Hz, 1H, β-pyrrolic H), 8.85 (d, *J* = 4.0 Hz, 1H, β-pyrrolic H), 8.92–8.95 (m, 4H, β-pyrrolic H), 9.30 (s, 1H, β-pyrrolic H). ESIMS *m*/*z*: [M + H]^+^ calcd for C_61_H_40_Cl_2_N_5_O_3_Zn, 1024.1794; found, 1024.1765; UV–vis (CH_2_Cl_2_) λ_max_: 403, 424, 552, 592 nm. Some crystals of C_61_H_39_Cl_2_N_5_O_3_Zn were recrystallized from CHCl_3_/*n*-hexane. A suitable crystal was selected and mounted on a Rigaku Saturn CCD area detector diffractometer. The crystal was kept at 113 K during data collection. Using Olex2 [54], the structure was solved with the XS [55] structure solution program using Direct Methods and refined with the olex2.refine [56] refinement package using Gauss–Newton minimisation. Crystal data for C_61_H_39_Cl_2_N_5_O_3_Zn (*M* = 1026.32 g/mol): monoclinic, space group *C*2/*c* (no. 15), *a* = 28.166(6) Å, *b* = 17.330(4) Å, *c* = 26.363(5) Å, β = 117.96(3)°, *V* = 11366(5) Å^3^, *Z* = 8, *T* = 113 K, μ(Mo Kα) = 0.573 mm^−1^, *Dcalc* = 1.1995 g/cm^3^, 40330 reflections measured (4.7° ≤ 2Θ ≤ 50.04°), 9924 unique (*R*_int_ = 0.0578, R_sigma_ = 0.0601) which were used in all calculations. The final *R*_1_ was 0.0861 (I>=2u(I)) and *wR*_2_ was 0.2556 (all data). The CCDC number of the crystal of **3a** is 1910675, which can be obtained free of charge from the Cambridge Crystallographic Data Centre via http://www.ccdc.cam.ac.uk/data_request/cif.

**3b**: Through the same procedure as that for **3a**, besides using 2,4,6-trimethylphenyl nitrile oxide [45], compound **3b** was obtained in a yield of 30% (36 mg). ^1^H NMR (500 MHz, CDCl_3_) δ 1.40 (s, 6H, CH_3_), 2.09 (s, 3H, CH_3_), 3.98 (s, 3H, CO_2_CH_3_), 4.42 (s, 1H, C-H), 6.29 (s, 1H, CH), 6.52 (s, 2H), 6.62 (t, *J* = 6.0 Hz, 1H), 6.95 (d, *J* = 7.5 Hz, 2H), 7.55 (t, *J* = 6.0 Hz, 1H), 7.69–7.77 (m, 10H), 7.82 (d, *J* = 7.0 Hz, 2H), 7.91(d, *J* = 7.5 Hz, 2H), 8.00 (d, *J* = 7.0 Hz, 1H), 8.56 (d, *J* = 7.0 Hz, 1H), 8.16–8.22 (m, 5H), 8.56 (d, *J* = 4.0 Hz, 1H, β-pyrrolic H), 8.84 (d, *J* = 4.0 Hz, 1H, β-pyrrolic H), 8.90–8.92 (m, 3H, β-pyrrolic H), 8.95 (d, *J* = 4.0 Hz, 1H, β-pyrrolic H), 9.19 (s, 1H, β-pyrrolic H); ESIMS *m*/*z*: [M + H]^+^ calcd for C_64_H_48_N_5_O_3_Zn, 998.3043; found, 998.3011; UV–vis (CH_2_Cl_2_) λ_max_: 402, 424, 552, 595 nm.

ZnTPP: The Zn-complex of TPP was prepared according to the literature [48], the yield was 89%. ^1^H NMR (500 MHz, CDCl_3_) δ 7.73–7.80 (m, 12H), 8.23 (dd, *J* = 7.5/1.4 Hz, 8H), 8.95 (s, 8H, β-pyrrolic H).

## Supporting Information

File 1 X-ray data of compound **3a**.
